# Skeletal Muscle Na,K-ATPase as a Target for Circulating Ouabain

**DOI:** 10.3390/ijms21082875

**Published:** 2020-04-20

**Authors:** Violetta V. Kravtsova, Elena V. Bouzinova, Vladimir V. Matchkov, Igor I. Krivoi

**Affiliations:** 1 Department of General Physiology, St. Petersburg State University, St. Petersburg 199034, Russia; violettakravtsova@gmail.com; 2 Department of Biomedicine, University of Aarhus, C 8000 Aarhus, Denmark; elena_bouz@hotmail.com (E.V.B.); vvm@biomed.au.dk (V.V.M.)

**Keywords:** skeletal muscle, Na,K-ATPase isozymes, ouabain, resting membrane potential

## Abstract

While the role of circulating ouabain-like compounds in the cardiovascular and central nervous systems, kidney and other tissues in health and disease is well documented, little is known about its effects in skeletal muscle. In this study, rats were intraperitoneally injected with ouabain (0.1–10 µg/kg for 4 days) alone or with subsequent injections of lipopolysaccharide (1 mg/kg). Some rats were also subjected to disuse for 6 h by hindlimb suspension. In the diaphragm muscle, chronic ouabain (1 µg/kg) hyperpolarized resting potential of extrajunctional membrane due to specific increase in electrogenic transport activity of the α2 Na,K-ATPase isozyme and without changes in α1 and α2 Na,K-ATPase protein content. Ouabain (10–20 nM), acutely applied to isolated intact diaphragm muscle from not injected rats, hyperpolarized the membrane to a similar extent. Chronic ouabain administration prevented lipopolysaccharide-induced (diaphragm muscle) or disuse-induced (soleus muscle) depolarization of the extrajunctional membrane. No stimulation of the α1 Na,K-ATPase activity in human red blood cells, purified lamb kidney and *Torpedo* membrane preparations by low ouabain concentrations was observed. Our results suggest that skeletal muscle electrogenesis is subjected to regulation by circulating ouabain via the α2 Na,K-ATPase isozyme that could be important for adaptation of this tissue to functional impairment.

## 1. Introduction

Na,K-ATPase is a vital transport protein that is ubiquitously expressed in the plasma membrane of all animal cells. The Na,K-ATPase is responsible for establishing and maintaining high K^+^ and low Na^+^ concentrations in the cytoplasm. This ion translocation activity underlies the resting membrane potential (RMP) as well as membrane excitability and provides the driving force for secondary ion transport [1]. In addition to its “classical” role in ion transport, the Na,K-ATPase is now considered as one of the most important signaling molecules in neuronal, epithelial, cardiac and vascular tissues [2,3,4].

The Na,K-ATPase is a heteromeric complex consisting of a catalytic and transport α subunit and glycoprotein β subunit. Four isoforms of the α subunit and three isoforms of the β subunit are expressed in a cell- and tissue-specific manner providing a wide molecular diversity of the Na,K-ATPase. In erythrocytes, kidney, lung and intestine the α1 isoform is predominantly expressed, while the majority of other cell types co-express the α1 isoform in a combination with other α isoforms. Thus, the α1 and α2 isoforms are co-expressed in skeletal, cardiac and smooth muscles as well as in glial cells, while the α1 and α3 isoforms are characteristic for neuronal tissues [5,6,7,8,9].

The extracellular loops of α subunit form a unique highly specific binding site for cardiotonic steroids (CTS), e.g., ouabain, marinobufagenin, digoxin and their circulating endogenous analogs [7,10,11,12,13,14]. The presence of endogenous ouabain-like compounds was suggested almost 40 years ago and endogenous ouabain was later purified from human blood plasma [10]. Ouabain is believed to be synthesized in the adrenal cortex and hypothalamus, however, the exact mechanisms and precursors involved in its biosynthesis are still unclear. Endogenous ouabain circulates normally at subnanomolar concentration range however, its elevated level has been reported under physiological conditions such as exercise [15,16] and pathophysiological conditions, e.g., congestive heart failure, hypertension and affective disorders [6,7,11,14]. It remains still uncertain whether binding of CTS affects cellular functions by inhibiting enzymatic activity altering ion homeostasis, or by conformational changes of the α subunit initiating signal transduction. 

Endogenous, circulating ouabain is suggested to be involved in numerous cell functions including gene expression, cell growth, survival and neuroprotection, differentiation, intercellular communications, inflammation, salt homeostasis and regulation of blood pressure, neural signaling and behavior [11,17,18,19,20]. Recently, a protective effect of ouabain against lipopolysaccharide (LPS) induced acute lung injury in mice was found [21]. While the unique role of circulating ouabain in cardiovascular and central nervous systems, kidney and other organs in health and disease is well documented, little is known about its role in skeletal muscle [16], containing one of the largest pools of Na,K-ATPase in the body.

This study examines the functional and expressional consequences of chronic ouabain administration on rat diaphragm and soleus muscles. We subjected rats to 4-day injections of ouabain alone and in a combination with LPS to induce acute injury; in some experiments, rats were also subjected to disuse for 6 h by hindlimb suspension (HS). LPS and HS interventions were used to test potential contribution of circulating ouabain to inflammatory and disuse responses. The following parameters were analyzed: the serum ouabain level and blood glucose level; the RMP of muscle fibers at different regions of the sarcolemma; the electrogenic transport activity of the α1 and α2 Na,K-ATPase and their mRNA expression and protein content. In addition, the concentration-dependent action of ouabain on different Na,K-ATPase preparations was examined.

## 2. Results

### 2.1. Ouabain and Glucose Levels Following Chronic Ouabain Treatment

**Figure 1:**
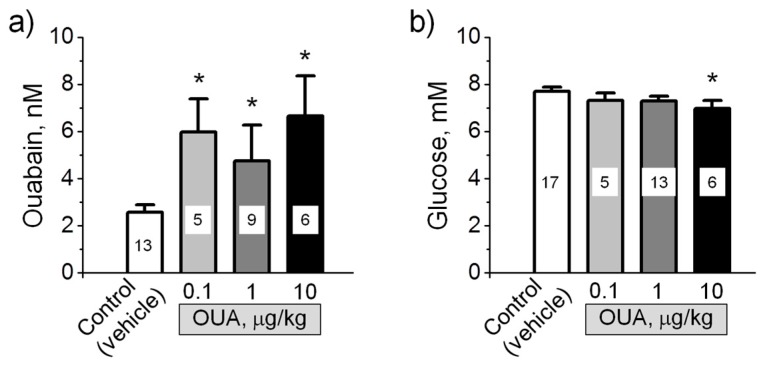
Serum ouabain level (**a**) and blood glucose level (**b**) of control rats and rats injected with different doses of ouabain (µg/kg, as indicated) for 4 days. The number of rats is indicated. * *p* < 0.05 compared with the corresponding control (vehicle treated group).

In accordance with previous observation [22], the serum level of ouabain in control rats was 2.6 ± 0.3 nM (Figure 1a). After the administration of exogenous ouabain in doses of 0.1–10 μg/kg, the serum level of ouabain significantly (*p* < 0.05) increased 1.8–2.6 times, supporting the efficiency of this protocol to elevate the level of circulating ouabain (Figure 1a). Blood glucose level was not changed by these ouabain injections, except ouabain in a dose of 10 μg/kg, which significantly (*p* < 0.05) reduced glucose level by approximately 10% (Figure 1b).

Notably, the level of circulating ouabain was not different while different ouabain doses were administered. The reasons for this discrepancy are not completely clear. Little is known about the form in which CTS circulates. CTS including ouabain are suggested to be transported as the complexes with protein-carrier(s) that provide a reservoir/buffer for CTS and protection from metabolism and renal clearance. Feedback mechanisms are suggested to participate in physiological regulation of the degree of CTS dissociation from its carrier and their circulating level [23,24,25].

### 2.2. Chronic Ouabain Differently Alters the Resting Membrane Potential in Distinct Sarcolemma Regions

**Figure 2:**
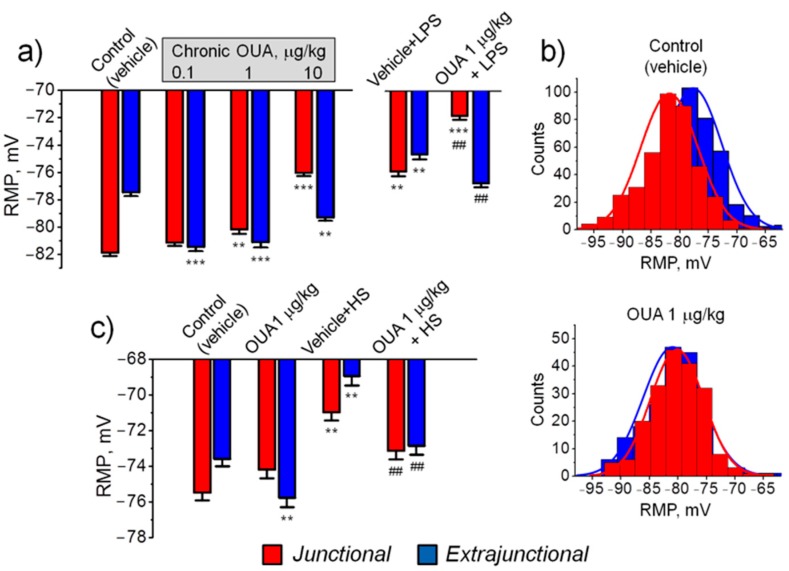
Effects of chronic ouabain (OUA) administration on the resting membrane potential (RMP) of rat diaphragm (**a,b**) and soleus (**c**) muscles. Rats were intraperitoneally injected with different doses of ouabain (µg/kg, as indicated) for 4 days. (**a**) Treatment with ouabain alone or with subsequent LPS (1 mg/kg) administration (see Methods). (**b**) The distributions of RMP in control and ouabain (1 µg/kg) treated muscles; the same data as in (**a**). (**c**) Treatment by ouabain (1 µg/kg) alone or with subsequent 6 h of hindlimb suspension (HS) (see Methods). The RMP reported in each data point represents the mean of measurements in at least 100 fibers from 4–6 diaphragm muscles and in at least 120 fibers from 6–8 soleus muscles. ** *p* < 0.01 and *** *p* < 0.001 compared with the corresponding control (vehicle treated group); ^##^ *p* < 0.01 compared with LPS- or HS-treated groups. Red – junctional; blue – extrajunctional membrane regions.

In the control (vehicle treated) diaphragm muscle the mean RMP of junctional (endplate) and extrajunctional membrane regions were −81.9 ± 0.3 mV and −77.5 ± 0.2 mV, respectively, i.e., the junctional region was significantly (*p* < 0.01) hyperpolarized with −4.4 ± 0.4 mV (Figure 2a) and distributions of RMP differed accordingly (Figure 2b). This local hyperpolarization is consistent with previous studies and is attributed to enhanced electrogenic activity of the α2 Na,K-ATPase isozyme in the endplate of rodents [26,27]. In the diaphragm muscle of rats treated with 0.1 µg/kg and 1 µg/kg ouabain for 4 days, hyperpolarization of the extrajunctional membrane was observed, reaching values of −4.0 ± 0.4 mV (*p* < 0.01) at 0.1 µg/kg ouabain treatment. The hyperpolarization was less with 10 µg/kg ouabain but was still significant (Figure 2a). Conversely, in the junctional region, only dose-dependent membrane depolarization was observed (Figure 2a). After chronic ouabain treatment, the local hyperpolarization of junctional membrane, observed in control, was absent and RMP distributions in junctional and extrajunctional membrane regions were not different (Figure 2b). These observations suggest an abnormal function of the Na,K-ATPase α2 isozyme in the endplate region.

In LPS-induced injury, chronic ouabain (1 µg/kg) completely prevented LPS-induced depolarization of the diaphragm extrajunctional membrane; in contrast, in the junctional membrane, ouabain pre-treatment only amplified LPS-induced depolarization (Figure 2a).

The first 6 h of HS is known to depolarize the rat soleus muscle sarcolemma [28]. In the soleus muscle, similar to diaphragm muscle, chronic ouabain (1 µg/kg) hyperpolarized only extrajunctional membrane (Figure 2c). Hovewer, ouabain pre-treatment prevented HS-induced depolarization in both extrajunctional and junctional membrane regions (Figure 2c).

**Figure 3:**
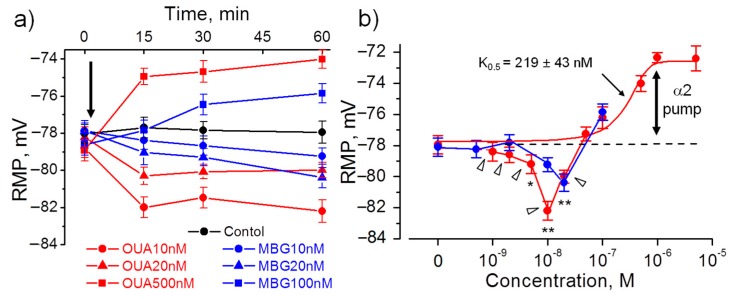
Acute hyperpolarizing and depolarizing effects of ouabain (OUA, red) and marinobufagenin (MBG, blue) at the extrajunctional membrane of diaphragm muscles obtained from non-treated rats. (**a**) The RMP dynamics at different OUA or MBG concentrations (as indicated). First RMP recording was made prior to addition of OUA or MBG (indicated by arrow). (**b**) RMP values after 60 min incubation with different OUA or MBG concentrations. The solid curve is fitted with Hill equation and triangles indicate data points excluded from this fit; the calculated inhibitory constant K_0.5_ for ouabain is indicated. Vertical arrow indicates the α2 Na,K-ATPase electrogenic contribution to the RMP. Each data point represents the mean of measurements in at least 100 fibers from 4–6 muscles. * *p* < 0.05 and ** *p* < 0.01 with the corresponding control (in the absence of ouabain or marinobufagenin).

Ouabain (10–20 nM) acutely added to isolated diaphragm muscles from non-treated rats also hyperpolarized the extrajunctional membrane to a similar extent: with −4.3 ± 0.8 mV (*p* < 0.01; for 10 nM concentration, 60 min incubation). Hyperpolarization fully developed after 15 min and remained stable for 60 min. At higher ouabain concentrations, only membrane depolarization was observed (Figure 3a,b). Ouabain depolarized muscle membrane in a dose–dependent manner that was best fitted with K_0.5_ = 219 ± 43 nM suggesting inhibition of the ouabain-sensitive α2 isoform of the Na,K-ATPase (Figure 3b).

Since rodent α1 isozyme is more then 100-fold less sensitive to ouabain binding, the ouabain-induced hyperpolarization is likely a result of stimulated electrogenic transport by the α2 Na,K-ATPase isozyme. This is in agreement with specific activation of the α2 isozyme in myocytes by nanomolar concentrations of ouabain [29].

Similar to ouabain, 10–20 nM marinobufagenin evoked hyperpolarization and depolarized membrane at higher concentrations; however, marinobufagenin changed RMP slower than ouabain (Figure 3a,b). Elevated levels of marinobufagenin are mostly known to associate with renal, cardiac and vascular dysfunctions [11,30,31]. Our data provide the evidence to suggest that marinobufagenin can also be considered as potential modulator of skeletal muscle electrogenesis.

### 2.3. Low Ouabain Concentrations Does Not Stimulate α1 Na,K-ATPase

**Figure 4:**
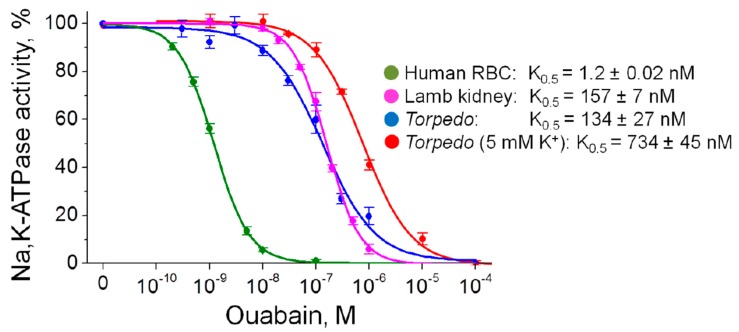
Inhibitory effects of ouabain on Na,K-ATPase activity in human red blood cells (RBC), purified lamb kidney and *Torpedo* membrane preparations. Incubation was performed with various concentrations of ouabain for 3 h in K^+^ free solutions; *Torpedo* membrane preparations were also incubated in the presence of 5 mM K^+^. The solid lines are a fit to the Hill equation; the corresponding calculated inhibitory constants K_0.5_ are indicated.

Although ouabain is a specific inhibitor of the Na,K-ATPase, the ability of ouabain to activate Na,K-ATPase at concentrations comparable to its endogenous level was shown. Ouabain is known to be able activate the α2 Na,K-ATPase [29] as well as the α1 Na,K-ATPase [32,33,34,35,36]. These data are still controversial and in other experiments on non-cellular Na,K-ATPase preparations, such effects were not observed [37,38,39]. In our study, no ouabain-induced stimulation of the Na,K-ATPase activity in human RBC, purified lamb kidney and *Torpedo* membrane preparations (expressing the α1 Na,K-ATPase isozyme only) was observed (Figure 4).

### 2.4. Chronic Ouabain Specifically Modulates α2 Na,K-ATPase Electrogenic Activity

**Figure 5:**
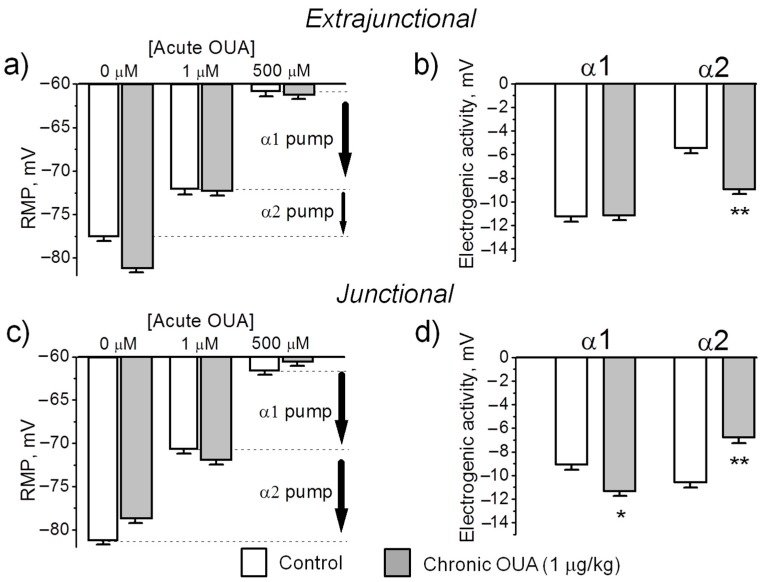
Chronic exposure to 1 µg/kg ouabain (OUA) for 4 days specifically alters the electrogenic transport by the α2 Na,K-ATPase isozyme. The RMP and α1 and α2 isozyme electrogenic activity were measured in the extrajunctional (**a**,**b**) and junctional (**c**,**d**) membrane regions of control (white bars) and ouabain-treated (grey bars) diaphragm muscles. The RMPs were recorded before and 30 min after incubation with 1 µM ouabain and 500 µM ouabain (see Methods). Vertical arrows indicate electrogenic contributions generated by the α1 and α2 Na,K-ATPase isozymes in control muscles. The RMP reported for each data point represents the mean of measurements of >150 fibers from muscles of 7 control and 8 ouabain-treated rats. * *p* < 0.05 and ** *p* < 0.01 compared with the corresponding control (vehicle treated group).

The transport activity of the Na,K-ATPase α1 and α2 isozymes was determined by measuring the ouabain-sensitive changes in RMP. This method is based on more than 100-fold higher affinity of the rodent α2 Na,K-ATPase isoform for ouabain compared to the α1 isoform. Ouabain was sequentially added at concentrations of 1 µM and 500 µM. For each muscle, the electrogenic contribution of α2 isozyme was computed as the difference of mean RMP before and 30 min after the incubation with 1 µM ouabain (Figure 3b shows the α2 isozyme electrogenic contribution inhibited by 1 µM ouabain). Then, the electrogenic contribution of α1 isozyme was estimated as the difference in RMP with 1 µM ouabain and after 30 min incubation with 500 µM ouabain (see Methods). Figure 5 shows the mean RMPs measured prior and after exposure to 1 µM and 500 µM ouabain. In the extrajunctional region of control muscles, total electrogenic activity by the Na,K-ATPase contributes to the RMP with –16.6 ± 0.4 mV. This contribution consists of −5.4 ± 0.5 mV from the α2 isozyme and −11.2 ± 0.4 mV generated by the α1 isozyme (Figure 5a,b). Chronic exposure to 1 µg/kg ouabain alters these contributions in an isoform-specific manner. The α2 isozyme contributed to electrogenic potential with −8.8 ± 0.4 mV (*p* < 0.01); while the electrogenic contribution from the α1 isozyme remained unchanged (Figure 5a,b). This suggests that chronic ouabain-induced membrane hyperpolarization is mediated by a specific increase in the α2 isozyme electrogenic activity.

In junctional membrane regions, the activity of α1 isozyme only slightly increased (*p* < 0.05) after chronic ouabain exposure. In contrast, α2 isozyme activity decreased from −10.5 ± 0.4 mV in control to −6.7 ± 0.5 mV (*p* < 0.01) after ouabain treatment (Figure 5c,d). This suggests that in junctional membrane regions, chronic ouabain produces depolarization mainly due to a specific decrease in the α2 isozyme activity.

Could changes in the RMP be caused by a mechanism other than a change in the Na,K-ATPase electrogenic activity? This possibility is unlikely due to the finding that both control and ouabain-treated muscles establish the same RMP (~ −61 mV) when the Na,K-ATPase electrogenic contribution is completely inhibited with 500 µM ouabain (Figure 5a,c). This result confirms that chronic ouabain treatment specifically alters the Na,K-ATPase activity without changing Nernst potential, which, in the absence of electrogenic transport, is solely determined by membrane permeability and ion gradients (Goldman-Hodgkin-Katz equation). Taken together, these data suggest that chronic ouabain changes the RMP via the α2 electrogenic pump contribution rather than by change in ion permeability.

### 2.5. Chronic Ouabain Modulates the α2 Na,K-ATPase Isozyme Electrogenic Activity without Changes in Protein Content

**Figure 6:**
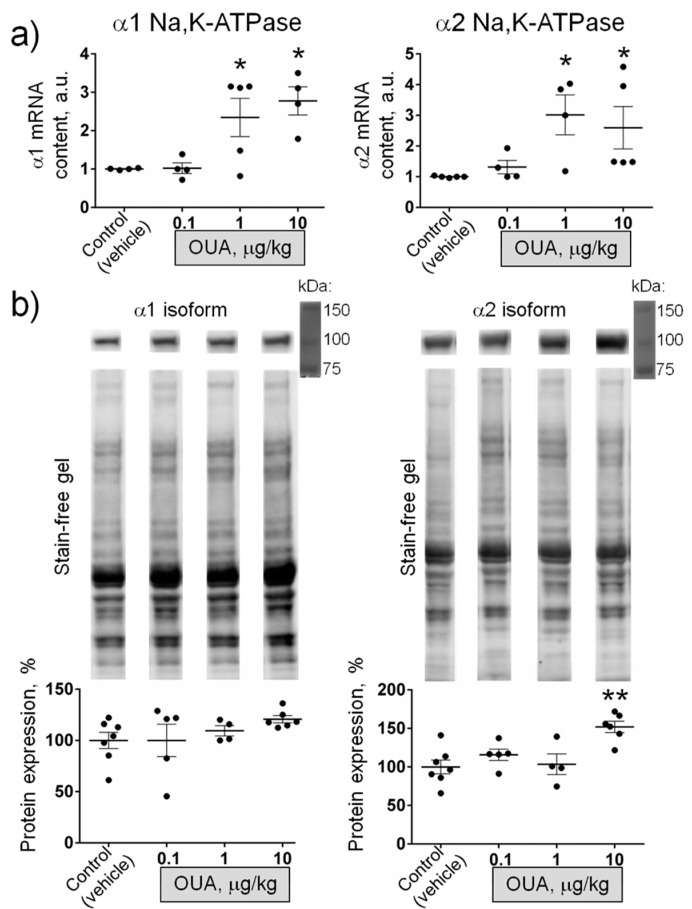
Chronic exposure to ouabain (OUA) specifically alters mRNA level and protein content of the α2 Na,K-ATPase in whole homogenate of diaphragm muscles. Rats were injected for 4 days with different doses (µg/kg) of ouabain as indicated. (**a**) Relative mRNA level for the α1 and α2 isoforms (*n* = 4–5). (**b**) Averaged Western blot analyses of α1 and α2 isoform protein expression (*n* = 4–7); representative Western blots for semi-quantification of the Na,K-ATPase α1 and α2 isoforms are shown. * *p* < 0.05 and ** *p* < 0.01 compared with the control (vehicle treated group).

Further, we tested if chronic exposure to ouabain modulates the α1 and α2 Na,K-ATPase mRNA and protein content measured in whole homogenates from diaphragm muscles. Chronic ouabain (0.1 µg/kg) did not significantly change the α1 Na,K-ATPase mRNA, while both α1 and α2 Na,K-ATPase mRNA were significantly (*p* < 0.05) increased in muscles from rats treated with 1 and 10 µg/kg ouabain (Figure 6a). The α1 Na,K-ATPase protein level was unchanged at all ouabain doses; the α2 Na,K-ATPase protein level was significantly (*p* < 0.01) increased only at 10 µg/kg ouabain (Figure 6b). 

These observations indicate that ouabain at doses of 0.1 and 1 µg/kg can modulate the RMP and the α2 Na,K-ATPase electrogenic activity without changes in α1 and α2 Na,K-ATPase protein level. Along with data on the ability of ouabain to hyperpolarize the membrane ex-vivo within 15 min (Figure 3a), it suggests that circulating ouabain acutely modulates skeletal muscle electrogenesis and this does not require any expressional changes.

## 3. Discussion

The α1 and α2 Na,K-ATPase isoforms are co-expressed in the skeletal muscles where the α2 isoform is the major α subunit. This isoform is essential for the contractile function and its activity and abundance can be regulated by skeletal muscle use [28,40,41,42]. Endogenous ouabain level also depends on skeletal muscle activity and is strongly enhanced during exercise [15,16]. Endogenous ouabain, associated with motor activity, has been shown to play a role in the adaptations to exercise, presumably, with the involvement of α2 Na,K-ATPase [16]. The novelty of our findings in this study is that: 1) skeletal muscle electrogenesis is a subject for regulation by circulating ouabain via the α2 Na,K-ATPase isozyme; 2) the RMP of distinct membrane regions (junctional and extrajunctional) are differently regulated by circulating ouabain; 3) circulating ouabain can modulate the RMP acutely without α2 Na,K-ATPase isozyme protein changes; 4) ouabain pre-treatment might be essential to prevent impaired sarcolemma electrogenesis.

There is a correlation between the amount/activity of Na,K-ATPase in skeletal muscle and glucose level [43]. Additionally, the α2 Na,K-ATPase is known to play a key role in metabolic functions of the body and its abundance is related to glucose metabolism [44]. The mechanism of this relationship is still unclear. In this study, chronic ouabain administration did not change blood glucose level, except relative high dose of 10 μg/kg that reduced glucose level by ~10% (Figure 1b). Thus, it is unlikely that the observed changes in the function of α2 Na,K-ATPase are due to changes in blood glucose level.

Maintaining the sufficient RMP is essential for many physiological processes, including ion homeostasis, excitability and the safety factor for neuromuscular transmission [27,45,46]. Steady membrane depolarization is characteristic for chronic motor dysfunction [42,47,48,49,50] when the α2 Na,K-ATPase is predominantly impaired in both animal models [48,49,50] and human [51,52]. Investigations into the early regulatory and signaling processes that precede overt skeletal muscle atrophy are needed to insight the molecular mechanisms of muscle remodeling during adaptations to disuse [28,53,54,55,56,57]. The loss of the α2 Na,K-ATPase isozyme electrogenic activity resulted in sarcolemma depolarization are observed in rat soleus muscle as early as 6 h of HS and these disturbances are among the earliest remodeling events induced by skeletal muscle disuse [28,53,54].

In this study, pre-treatment with low doses (1 µg/kg) of ouabain prevented HS-induced depolarization of rat soleus muscle sarcolemma (Figure 2c). LPS, administrated to induce acute injury, also depolarized diaphragm muscle sarcolemma. However, ouabain (1 µg/kg) pre-treatment completely prevented the depolarization of the extrajunctional membrane region, while in the junctional membrane only amplified LPS-induced depolarization (Figure 2a). Thus, the findings of this study provide the first evidence that circulating ouabain could be important for adaptation of skeletal muscle electrogenesis to functional impairment.

It was previously shown that chronic, but not acute, intraperitoneal administration of a low dose (1 µg/kg) of ouabain significantly improves mouse recovery following traumatic brain injury [18]. In this study, ouabain at the doses of 0.1 µg/kg and 1 µg/kg modulates the α2 Na,K-ATPase electrogenic activity and the RMP without changes in α2 Na,K-ATPase protein content in whole homogenates from diaphragm muscles. Moreover, ouabain at nanomolar concentrations was able acutely (within 15 min) hyperpolarize the extrajunctional membrane (Figure 3a). These observations suggest that circulating ouabain acutely modulates skeletal muscle electrogenesis and this does not require any expressional changes. Further experiments are required to validate this possibility. 

Notably, our study suggests that distinct membrane regions (junctional and extrajunctional) (Figure 2a,c) as well as corresponding α2 Na,K-ATPase membrane pools (Figure 5b,d) are differently regulated by circulating ouabain. The reason for this difference remains unclear. External K^+^ is known to antagonize ouabain binding to the Na,K-ATPase [58]. Two main pools of the α2 Na,K-ATPase are present in skeletal muscles. The majority of α2 isozyme is expressed in the interior T-tubule membranes [41] and the smaller α2 isozyme pool is localized to the junctional membrane [26,28]. Since K^+^ is known to accumulate in both synaptic clefts [59] and T-tubules [41], it can be suggested that depending on K^+^ accumulation, circulating ouabain can differently modulate these distinct pools of the α2 Na,K-ATPase. This may be the reason for opposite effects of chronic ouabain in extrajunctional and junctional membrane regions observed in this study (Figure 2a,c).

Our study suggests that the extrajunctional pool of α2 Na,K-ATPase is activated by circulating ouabain. This corresponds to greater ouabain sensitivity of the α2 isoform compared with the α1 isoform in rodents as well as to the ouabain-induced α2 Na,K-ATPase activation in cardiomyocytes of different species [29]. The ability of ouabain to activate the Na,K-ATPase at concentrations comparable to its endogenous level is well known, however, the mechanism has been a subject of debate ever since. This stimulation was suggested to be a result of direct action of low CTS concentrations and the existence of two ouabain-binding sites with high (stimulatory) and low affinities (inhibitory) was assumed [29]. The presence of second binding site in the same α subunit still debated [60,61]. The existence of Na,K-ATPase in a form of (αβ)_2_ diprotomer with functionally different α subunits [62] having different ouabain affinities is also discussed regarding the α1 subunit in endothelial cells [35]. It is unknown whether this mechanism is present in skeletal muscle. Moreover, low ouabain concentration did not show any direct stimulation of the α1 Na,K-ATPase in this study (Figure 4) and other reports [37,38,39].

Alternatively, ouabain-mediated stimulation of the α1 Na,K-ATPase in renal cells requires specific molecular environment, such as sodium/hydrogen exchanger-1 [33] or angiotensin receptor type I, and can be modulated by the initial increase in intracellular concentration of Na^+^ [34]. This Na^+^ accumulation, triggered by endogenous ouabain, is suggested to enhance translocation of the Na,K-ATPase from intracellular pool to plasma membrane through an angiotensin/AT1R-dependent mechanism [34]. However, these signaling are expected to occur in a time scale longer than the acute hyperpolarization seen in this study (15 min, Figure 3a) and, thus, cannot explain our findings.

One can assume that accumulated Na^+^ can immediately activate ouabain-free neighboring Na,K pumps. To operate in such manner, ouabain should induce Na^+^ accumulation in the subcellular micro-compartments similar to “PLasmERosome” model [20]. In the skeletal muscle, an analogue of this microdomain can be triadic junctions formed by T-tubules and terminal cisternae of the sarcoplasmic reticulum, where the α2 Na, K-ATPase [41] and the Na^+^,Ca^2+^ exchanger [63] are localized. This localization could also explain the hyperpolarizing effects of chronic ouabain, observed only in the extrajunctional membrane region (Figure 2a,c).

## 4. Materials and Methods 

### 4.1. Animals

Experiments were performed on male Wistar rats (180–230 g). Animals were housed in a temperature- and humidity-controlled room with food and water ad libitum. All procedures involving rats were performed in accordance with the recommendations for the Guide for the Care and Use of Laboratory Animals [64]. The experimental protocol met the requirements of the EU Directive 2010/63/EU for animal experiments and was approved by the Ethics Committee of St. Petersburg State University (issued 13 December 2017) and the Animal Experiments Inspectorate of the Danish Ministry of Environment and Food (issued 5 July 2016).

Rats were intraperitoneally injected with vehicle (0.9% NaCl) or 0.1, 1 and 10 µg/kg body weight ouabain once daily for 4 days as described previously [33]. In some experiments, two hours after last injection of ouabain (1 µg/kg), lipopolysaccharide (LPS, 1 mg/kg) was intraperitoneally administrated to induce acute injury. Twenty-four hours after last injection of ouabain, diaphragm muscles were isolated. In separate experiments, twenty-four hours after the last ouabain (1 µg/kg) injection, rats were subjected to HS, widely used as an animal model of disuse that leads to progressive atrophy of postural skeletal muscles. The rats were subjected to HS individually in custom cages for 6 h, as described previously [65]. Control animals were not suspended. In these experiments, soleus muscles were isolated. Freshly isolated diaphragm or soleus muscles were immediately used for electrophysiological experiments. For later biochemical assays, some diaphragm muscles were snap-frozen in liquid nitrogen and then stored at −80 °C.

In a separate set of experiments, ouabain or marinobufagenin at different concentrations were acutely added to isolated intact diaphragm muscles obtained from non-treated rats.

### 4.2. Ouabain and Glucose Level Measurements

The serum level of ouabain was estimated using ELISA Kit for Ouabain (Cloud-Clone corp., Katy, TX, USA). The blood glucose level was measured by applying a drop of blood to chemically treated, disposable “test-strip”, which was then inserted into electronic blood glucose meter (Accu-Chek Active, Roche Diabetes Care GmbH, Mannheim, Germany).

### 4.3. Membrane Potential Recording

The isolated muscle with nerve stump was placed in a chamber and continuously perfused with physiological solution containing (in mM): NaCl, 137; KCl, 5; CaCl_2_, 2; MgCl_2_, 2; NaHCO_3_, 24; NaH_2_PO_4_, 1; glucose, 11; pH 7.4. The solution was continuously gassed with 95% O_2_ and 5% CO_2_ and maintained at 28 °C. The RMPs were recorded from the surface fibers using intracellular glass microelectrodes. The RMP recordings were made in extrajunctional membrane regions within ~2 mm from visually identified terminal branches of the nerve, or directly near the nerve terminals, as described previously [26,66]. In each muscle, RMPs were recorded from 25–35 different fibers for each (junctional and extrajunctional) membrane region over a total time of about 5–10 min.

### 4.4. Measurement of Na,K-ATPase Electrogenic Activity in Intact Muscle 

Na,K-ATPase electrogenic transport was determined in intact muscle by measuring the ouabain-sensitive changes in RMP. These changes are generated by electrogenic Na,K-ATPase transport and are sensitive, real-time assay to assess the Na,K-ATPase activity in intact skeletal muscle [26,47,67]. This method is based on more than 100-fold difference in affinities of the rodent α1 and α2 Na,K-ATPase isoforms for ouabain. Thus, in rat skeletal muscle 1 μM ouabain inhibits the α2 isoform without affecting the α1 isoform, whereas 500 μM ouabain completely inhibits both isoforms [26,67]. The electrogenic contribution of α2 isozyme was computed as the difference in mean RMP before and 30 min after the incubation with 1 µM ouabain. The electrogenic contribution of α1 isozyme was estimated as the difference in RMP with 1 µM ouabain and after 30 min incubation with 500 µM ouabain.

### 4.5. Dose-Response Ouabain Effects in Different Na,K-ATPase Preparations

Transport activity of the Na,K-ATPase was determined by ouabain-sensitive influx of non-radioactive Rb^+^ into human red blood cells (RBC) using emission flame photometry as described [66,68]. RBC were obtained from 2 mL of whole blood, washed with 4-fold volume of cold (4 °C) solution consisting of 145 mM NaCl and 10 mM TRIS (pH 7.4), and centrifuged at 600 g for 3 min. The supernatant and upper layer containing leukocytes were removed and the RBC washed again. This procedure was repeated 4 times prior the precipitated RBC were diluted back to 2 mL by an incubation solution containing (in mM): NaCl, 145; CaCl_2_, 1; NaH_2_PO_4_, 1; MgCl_2_, 2; TRIS, 10; glucose, 10; pH 7.4. Each sample consisted of 0.1 mL of RBC suspension diluted with 0.9 mL of the incubation solution. The RBC were incubated with various concentrations of ouabain for 3 h at 37 °C. Then, 0.1 mL of 50 mM RbCl was added to all samples and incubation was continued for an additional 30 min. Subsequently, the samples were centrifuged for 3 min at 800 g and washed 4 times with a 4°C solution of 93 mM MgCl_2_. The precipitated RBC were hemolysed by 1 mL of distilled water added to each sample. The samples were then stirred and kept for 24 h at 4 °C. Concentrations of Rb^+^, K^+^ and Na^+^ were measured using a Perkin Elmer 306 atomic absorption spectrophotometer. The transport activity of the Na,K-ATPase was estimated as the difference between Rb^+^ influx in the presence and absence of 1 mM ouabain.

The activity of the Na,K-ATPase in membrane preparations from electric organ of *Torpedo californica* (a gift from Dr. Steen Pedersen, Baylor College of Medicine, TX, USA) or the activity of purified Na,K-ATPase from lamb kidney (a gift from Dr. W. Ball, University of Cincinnati Medical Center, OH, USA) was estimated using the coupled Pyruvate Kinase/Lactic dehydrogenase linked-enzyme system as described previously [69]. Incubation solution contained (in mM): L-histidine, 45; MgCl_2_, 10; NaCl, 100; phosphoenol pyruvate, 1; β-NADH, 0.45; ATP, 5; pyruvate kinase-lactic dehydrogenase–15 µL/1.25 mL of buffer solution (pH 7.3). Incubation was performed with various concentrations of ouabain for 3 h at 37 °C (the Na,K-ATPase from lamb kidney) or at room temperature (*Torpedo* membrane preparations). The decrease of NADH absorbance was measured at 340 nM (Beckman DU-7 spectrophotometer; Beckman Coulter Inc., Brea, CA, USA). The specific Na,K-ATPase activity was estimated as the difference between activity in the presence and absence of 1 mM ouabain (10 min pre-incubation).

Each data point was obtained as a result of measuring the activity of Na,K-ATPase in 3–4 experiments in triplets.

### 4.6. Western Blot Assays

Muscle was lysed in lysis buffer (in mM: Tris-HCl 10, sucrose 250, EDTA 1, EGTA 1, Triton X-100 2%, pH 7.4; and 1 tablet protease inhibitor per 10 ml). The homogenate was centrifuged at 10,000× *g*. Total protein concentrations in the supernatants were measured using BCA Protein Assay Kit (Thermo Fisher Scientific, Waltham, MA, USA). Ten micrograms of total protein diluted in Laemmli sample buffer (Bio-Rad, Hercules, CA, USA) were loaded to on 4–20% precast polyacrylamide stain-free gels (CriterionTM TGX Stain-freeTM precast gel, BioRad, Hercules, CA, USA). Total protein load was detected on the stain-free gels using UV-light in imaging system (c600, Azur Biosystems Inc., Dublin, CA, USA). The protein were electrotransferred to membranes that were then blocked by an incubation in 5% bovine serum albumin and 5% nonfat dry milk in PBS with 0.5% *v*/*v* Tween 20 (PBS-T). The membranes were incubated overnight at 5 °C with either α1 isoform Na,K-pump antibody (monoclonal, HPR-conjugated, 1:2000; Novus Biologicals Inc., Centennial, CO, USA) or with antibody against the α2 isoform (1:2000, Merck Millipore, Burlington, MA, USA). After intensive washing, the membranes were incubated with horseradish-peroxidase (HRP)-conjugated secondary antibody (1:4000; Dako Agilent, Santa Clara, CA, USA) for 1 h in PBS-T. Excess antibody was removed by washing, and bound antibody was detected by an enhanced chemiluminiscence kit (ECL, Amersham, Little Chalfont, UK). Detected protein was normalized using the ImageJ program (NIH, Bethesda, MD, USA) as a ratio to total protein load measured for the same probe.

### 4.7. Quantitative Polymerase Chain Reaction (PCR)

Isolated muscles were mechanically disrupted in Tissue Lyser (Qiagen, Hilden, Germany). The RNA isolation was done with Qiagen mini kit (Qiagen, Hilden, Germany). The reaction was executed with reverse transcriptase III (Invitrogen, Carlsbad, CA, USA) and superase (Ambion Ltd., Austin, TX, USA) for deactivation of RNAse and DNAse. Primer sets for quantitative PCR analyses of the α1 and α2 isoforms of Na,K-ATPase, S18 and Glyceraldehyde 3-phosphate dehydrogenase (GAPDH) expression were obtained from Applied Biosystems (Thermo Fisher Scientific, Waltham, MA, USA). Quantitative PCR was carried out on MX3000P (Stratagene, San Diego, CA, USA) using Taqman probe (FAM) technology. The expression of α1 and α2 isoforms was normalized to GAPDH and S18 (average Ct value) gene expression and presented as ΔCt value. ΔΔCt for averaged control muscles and studied sample were used to compare the expression of gene of interest, thereby standardized to control muscles. Relative gene expression was calculated as 1/(2ΔΔ*C*t) [70].

### 4.8. Materials

Ouabain, LPS and other chemicals were purposed from Sigma-Aldrich. Marinobufagenin was a gift from Dr. Nikolai Kolodkin (Institute of Highly Pure Biopreparations, St. Petersburg, Russia).

### 4.9. Statistics

All data are given as the mean ± SEM. Statistical significance of the difference between means was evaluated using a Student’s t-test and one-way ANOVA. Statistical analysis was performed using GraphPad Prism 7 software (GraphPad; San Diego, CA, USA). A probability value of *p* < 0.05 was considered statistically significant.

## 5. Conclusions

The overall conclusion of the present study is that skeletal muscle electrogenesis can be functionally regulated by circulating ouabain with specific involvement of the α2 Na,K-ATPase. These effects may have a profound impact and endogenous ouabain might be an important player in adaptations of skeletal muscle to functional impairment. The mechanisms of regulatory effects of circulating ouabain in skeletal muscle remain to be elucidated. Further studies are necessary to identify precise molecular basis of our findings and their functional significance. This knowledge could be useful in order to establish possible perspective of ouabain-like modulators of the α2 Na,K-ATPase for new effective therapeutic strategy.

## Abbreviations

CTS	cardiotonic steroids
HS	hindlimb suspension
LPS	lipopolysaccharide
MBG	marinobufagenin
OUA	ouabain
RBC	red blood cells
RMP	resting membrane potential
